# Supplementary material for: Immunogenicity and protective efficacy of SARS-CoV-2 mRNA vaccine encoding secreted non-stabilized spike in female mice
Source: Nat Commun. 2023 Apr 21;14:2309. doi: 10.1038/s41467-023-37795-0 (PMC10120480; doi:10.1038/s41467-023-37795-0)
Supplement: Supplementary file 1 — Supplementary Information [file 41467_2023_37795_MOESM1_ESM.pdf]

Supplement data 1

1. SARS-CoV-2 Spike (S) ectodomain amino acid sequence

MFVFLVLLPLVSSQCVNLTTRTQLPPAYTNSFTRGVYYPDKVFRSSVLHSTQDLFLPFFSNV
TWFHAIHVSGTNGTKRFDNPVLPFNDGVYFASTEKSNIIRGWIFGTTLDSKTQSLLIVNNATN
VVIKVCEFQFCNDPFLGVYYHKNNKSWMESEFRVYSSANNCTFEYVSQPFLMDLEGKQGN
FKNLREFVFKNIDGYFKIYSKHTPINLVRDLPQGFSALEPLVDLPIGINITRFQTLLALHRSYLT
PGDSSSGWTAGAAAYYVGYLQPRTFLLKYNENGTITDAVDCALDPLSETKCTLKSFTVEKGI
YQTSNFRVQPTESIVRFPNITNLCPFGEVFNATRFASVYAWNRKRISNCVADYSVLYNSASF
STFKCYGVSPTKLNDLCFTNVYADSFVIRGDEVRQIAPGQTGKIADYNYKLPDDFTGCVIAW
NSNNLDSKVGGNYNYLYRLFRKSNLKPFERDISTEIYQAGSTPCNGVEGFNCYFPLQSYGF
QPTNGVGYQPYRVVVLSFELLHAPATVCGPKKSTNLVKNKCVNFNFNGLTGTGVLTESNKK
FLPFQQFGRDIADTTDAVRDPQTLEILDITPCSFGGVSVITPGTNTSNQVAVLYQDVNCTEV
PVAIHADQLTPTWRVYSTGSNVFQTRAGCLIGAEHVNNSYECDIPIGAGICASYQTQTNSPR
RARSVASQSIIAYTMSLGAENSVAYSNNSIAIPTNFTISVTTEILPVSMTKTSVDCTMYICGDS
TECSNLLLQYGSFCTQLNRALTGIAVEQDKNTQEVFAQVKQIYKTPPIKDFGGFNFSQILPDP
SKPSKRSFIEDLLFNKVTLADAGFIKQYGDCLGDIAARDLICAQKFNGLTVLPPLLTDEMIAQY
TSALLAGTITSGWTFGAGAALQIPFAMQMAYRFNGIGVTQNVLYENQKLIANQFNSAIGKIQ
DSLSSTASALGKLQDVVNQNAQALNTLVKQLSSNFGAISSVLNDILSRLDKVEAEVQIDRLIT
GRLQSLQTYVTQQLIRAAEIRASANLAATKMSECVLGQSKRVDFCGKGYHLMSFPQSAPH
GVVFLHVTYVPAQEKNFTTAPAICHDGKAHFPREGVFVSNGTHWFVTQRNFYEPQIITTDN
TFVSGNCDVVIGIVNNTVYDPLQPELDSFKEELDKYFKNHTSPDVDLGDISGINASVVNIQKE
IDRLNEVAKNLNESLIDLQELGKYEQYI

Supplementary Table 1: Characterization of encapsulated mRNA and stability testing

**Storage Time**	**1-month**			**6-month**		**12-month**	
**Storage Temperature**	(-75 °C ± 10 °C)	(-20 °C ± 5 °C)	(5 °C ± 3 °C)	(-75 °C ± 10 °C)	(-20 °C ± 5 °C)	(-75 °C ± 10 °C)	(-20 °C ± 5 °C)
**Tested Parameters**							
mRNA encapsulation efficiency	97%	97%	95%	97%	95%	97%	95%
Particle Size (nm)	64 nm	64 nm	66 nm	65 nm	78 nm	65 nm	92 nm
Polydispersity Index (PDI)	0.05	0.06	0.07	0.08	0.10	0.06	0.14

Supplementary Figure 1

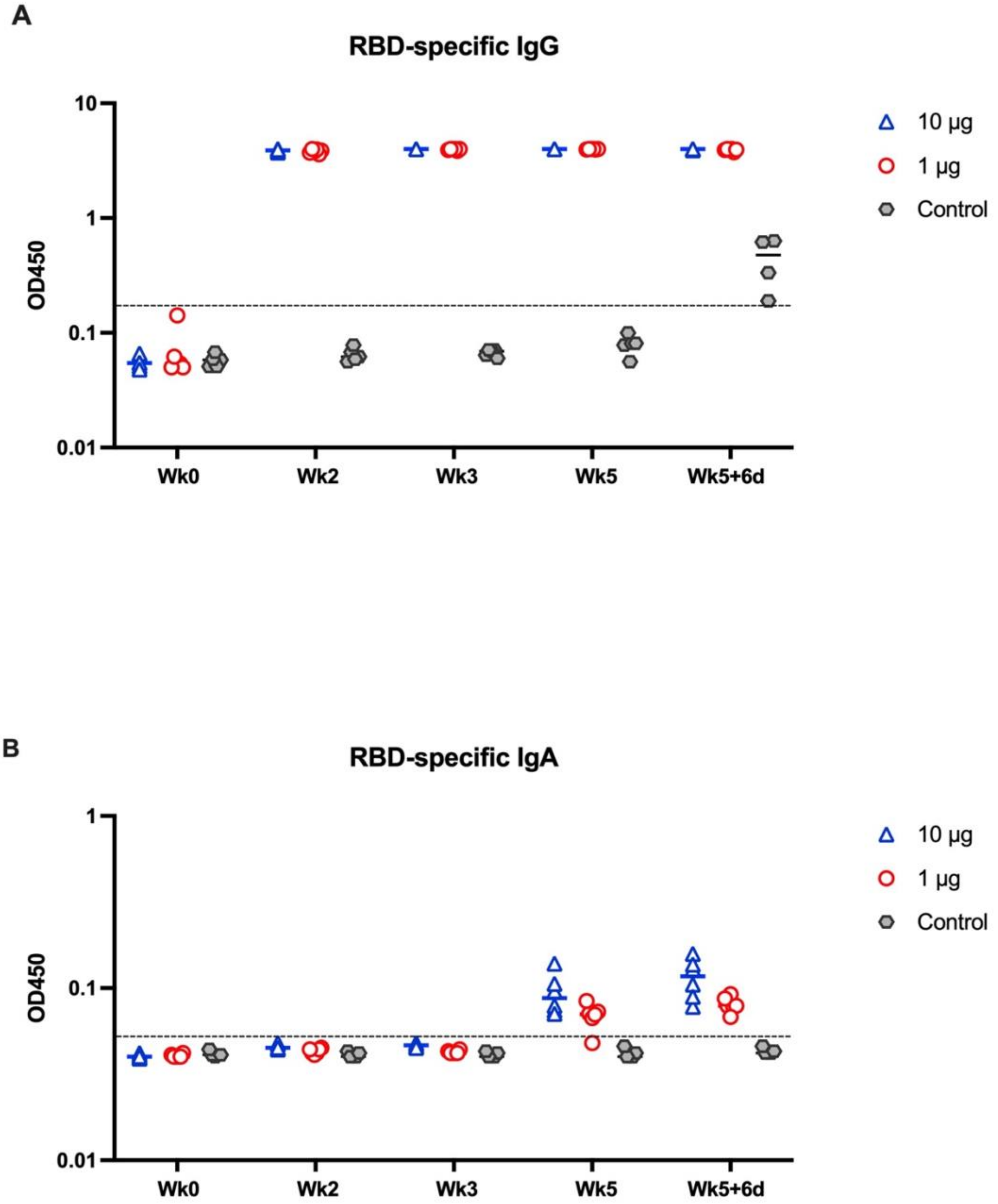

Figure S1: RBD-specific-IgG and -IgA from K18-hACE2 mice sera in the challenge experiment analyzed by ELISA at various timepoints. Sera were diluted 1:100 and measured for RBD-specific-IgG (A) and -IgA (B). OD450 results of individual mice immunized with 10 µg (n = 6, blue triangle), 1 µg (*n* =6, red circle) and PBS control (n = 5, grey hexagonal) were shown. Horizontal lines represent a positive cut-off.
