# Supplementary material for: Immunogenicity and protective efficacy of SARS-CoV-2 mRNA vaccine encoding secreted non-stabilized spike in female mice
Source: Nat Commun. 2023 Apr 21;14:2309. doi: 10.1038/s41467-023-37795-0 (PMC10120480; doi:10.1038/s41467-023-37795-0)
Supplement: Supplementary file 3 — Reporting Summary [file 41467_2023_37795_MOESM3_ESM.pdf]

nature portfolio

Corresponding author(s): Chutitorn Ketloy

Last updated by author(s): February 19, 2023

Reporting Summary

Nature Portfolio wishes to improve the reproducibility of the work that we publish. This form provides structure for consistency and transparency in reporting. For further information on Nature Portfolio policies, see our Editorial Policies and the Editorial Policy Checklist.

Statistics

For all statistical analyses, confirm that the following items are present in the figure legend, table legend, main text, or Methods section.

n/a	Confirmed	
☐	☒	The exact sample size (n) for each experimental group/condition, given as a discrete number and unit of measurement
☐	☒	A statement on whether measurements were taken from distinct samples or whether the same sample was measured repeatedly
☐	☒	The statistical test(s) used AND whether they are one- or two-sided<br>*Only common tests should be described solely by name; describe more complex techniques in the Methods section.*
☒	☐	A description of all covariates tested
☒	☐	A description of any assumptions or corrections, such as tests of normality and adjustment for multiple comparisons
☐	☒	A full description of the statistical parameters including central tendency (e.g. means) or other basic estimates (e.g. regression coefficient) AND variation (e.g. standard deviation) or associated estimates of uncertainty (e.g. confidence intervals)
☐	☒	For null hypothesis testing, the test statistic (e.g. F, t, r) with confidence intervals, effect sizes, degrees of freedom and P value noted<br>*Give P values as exact values whenever suitable.*
☒	☐	For Bayesian analysis, information on the choice of priors and Markov chain Monte Carlo settings
☒	☐	For hierarchical and complex designs, identification of the appropriate level for tests and full reporting of outcomes
☒	☐	Estimates of effect sizes (e.g. Cohen's d, Pearson's r), indicating how they were calculated

Our web collection on statistics for biologists contains articles on many of the points above.

Software and code

Policy information about availability of computer code

Data collection	Immunofluorescent imaging was performed using confocal microscope (ZEISS LSM 800, Carl Zeiss.<br>SkanIt version 2.4.5 or Magellan programs were use for microplate reader,<br>CTL ImmunoSpot S6 Macro M2 was used for ELISpot reading
Data analysis	Neutralizing Ab titers were analyzed by SPSS Statistics version 22<br>Graphs plotting and data analysis were performed Prism software version 9.4.1

For manuscripts utilizing custom algorithms or software that are central to the research but not yet described in published literature, software must be made available to editors and reviewers. We strongly encourage code deposition in a community repository (e.g. GitHub). See the Nature Portfolio guidelines for submitting code & software for further information.

Data

Policy information about availability of data

All manuscripts must include a data availability statement. This statement should provide the following information, where applicable:

- Accession codes, unique identifiers, or web links for publicly available datasets
- A description of any restrictions on data availability
- For clinical datasets or third party data, please ensure that the statement adheres to our policy

Accession code: The SARS-CoV-2 spike (S) gene, MN908947.1

https://www.ncbi.nlm.nih.gov/nuccore/MN908947.1

The data supporting the findings of this work are available within the paper and in the Supplementary Information file. Source data are provided as a source data file. Source data are provided with this paper.

Human research participants

Policy information about studies involving human research participants and Sex and Gender in Research.

Reporting on sex and gender	N/A
Population characteristics	N/A
Recruitment	N/A
Ethics oversight	N/A

Note that full information on the approval of the study protocol must also be provided in the manuscript.

Field-specific reporting

Please select the one below that is the best fit for your research. If you are not sure, read the appropriate sections before making your selection.

☒ Life sciences ☐ Behavioural & social sciences ☐ Ecological, evolutionary & environmental sciences

For a reference copy of the document with all sections, see nature.com/documents/nr-reporting-summary-flat.pdf

Life sciences study design

All studies must disclose on these points even when the disclosure is negative.

Sample size	No sample size calculations were used to power each study. Sample size for the mouse studies were determined based upon previous experience with similar studies where 5-7 animals per group represented a sufficient sample size to detect statistical differences between experimental groups [REFs to PMID: PMID: 33750975 and PMID: 35890321]. Sample size was also limited by space available in an animal ABSL-3 containment facility.
Data exclusions	No data was excluded
Replication	Elisa and neutralization assay were performed at least by duplication with similar results.
Randomization	All animals were randomly allocated to the different groups by similarity of age.
Blinding	The technicians performing the assays were blinded to group association and groups of samples were analyzed without knowledge of their origin, except for histological study in challenge test.

Reporting for specific materials, systems and methods

We require information from authors about some types of materials, experimental systems and methods used in many studies. Here, indicate whether each material, system or method listed is relevant to your study. If you are not sure if a list item applies to your research, read the appropriate section before selecting a response.

Materials & experimental systems

n/a	Involved in the study	
☐	☒	Antibodies
☐	☒	Eukaryotic cell lines
☒	☐	Palaeontology and archaeology
☐	☒	Animals and other organisms
☒	☐	Clinical data
☒	☐	Dual use research of concern

Methods

n/a	Involved in the study	
☒	☐	ChIP-seq
☒	☐	Flow cytometry
☒	☐	MRI-based neuroimaging

Antibodies

Antibodies used	Mouse monoclonal-anti-RBD (Clone 1034522, Cat No MAB10540, R&D Systems, Lot CMDN0120083), Immunofluorescence dilution 1:200, Western blot dilution 1:2,500.<br>Rabbit polyclonal-anti-S1(Cat No 40150-T62-COV2, Sino Biological, Lot HD14JN0901), Immunofluorescence dilution 1:200, Western blot dilution 1:5,000.<br>Rabbit polyclonal-anti-S2 (Cat No 40590-T62, Sino Biological, Lot HD14JU1604), Immunofluorescence dilution 1:200, Western blot dilution 1:5,000.<br>Pooled convalescent serum (PCS) collected in 2020, dilution 1:5,000.<br>Goat-anti-mouse IgG-FITC (Cat No 11-4011-85, BioLegend, Lot 1970160), dilution 1:5,000<br>Donkey-anti-rabbit IgG-FITC (Cat No 406403, BioLegend, Lot B283712), dilution 1:5,000<br>Goat-anti-human IgG AlexaFluor647 (Cat No 2048-31, Southern Biotech), dilution 1:5,000<br>Goat-anti-human IgG-HRP, (Cat No 5220-0330 , KPL, USA), dilution 1:10,000<br>Goat-anti-mouse IgG-HRP, (Cat No 5220-0341 , KPL, USA), dilution 1:10,000<br>Goat-anti-rabbit IgG-HRP, Cat No 5220-0336 , KPL, USA), dilution 1:10,000<br>Goat anti-mouse IgG (H+L)-HRP (Cat No 5450-0011, KPL, Lot 10311576), Dilution 1:10,000<br>Goat anti-mouse IgG1-HRP (Cat No 1070-05, Southern Biotech, Lot A5919-P199D), Dilution 1:5,000<br>Goat anti-mouse IgG2a-HRP (Cat No 1080-05, Southern Biotech, Lot L2618-MN69B), Dilution 1:5,000<br>Rabbit monoclonal-anti-Nucleoprotein (Clone 001, Cat No 40143-R001, Sino Biological), Dilution 1:5,000<br>Goat anti-rabbit-HRP-conjugated IgG secondary (AB2617138, Cat No P0448, Dako, Lot 41372205), Dilution 1:2,000<br>Rat-anti-mouse-IFN-γ-biotinylated mAb (Clone R4-6A2, Cat No 3321-6-1000, Mabtech), Dilution 1:2,500<br>Goat anti-mouse IgG (H+L)-HRP (Cat No 5220-0330, KPL, USA), Dilution 1:40,000<br>Goat anti-mouse IgA-HRP (Cat No 5220-0360, KPL, USA), Dilution 1:10,000
Validation	All primary antibodies were validates by the manufacturer, details as follows:<br>Mouse monoclonal-anti-RBD (Clone 1034522, Cat No MAB10540, R&D Systems, Lot CMDN0120083), https://www.rndsystems.com/products/sars-cov-2-spike-rbd-antibody-1034522_mab10540.<br>Rabbit polyclonal-anti-S1 (Cat No 40150-T62-COV2, Sino Biological, Lot HD14JN0901), https://www.hoelzel-biotech.com/media/import/pdf_pds/Sino_Biological/40150-T62-COV2-50__PDS.pdf<br>Rabbit polyclonal-anti-S2 (Cat No 40590-T62, Sino Biological, Lot HD14JU1604),<br>https://www.sinobiological.com/antibodies/cov-spike-40590-t62<br>Rabbit monoclonal-anti-Nucleoprotein (Clone 001, Cat No 40143-R001, Sino Biological), Dil https://www.sinobiological.com/antibodies/cov-nucleocapsid-40143-r001<br>Rat-anti-mouse-IFN-γ-biotinylated mAb (Clone R4-6A2, Cat No 3321-6-1000, Mabtech), https://www.mabtech.com/api/files/product_datasheets/3321-6-250.pdf

Eukaryotic cell lines

Policy information about cell lines and Sex and Gender in Research

Cell line source(s)	African green monkey kidney epithelial cells (Vero-E6) were from ATCC CRL-1586™<br>HEK293 cells were from ATCC CRL-3216™ for production of HEK293T-hACE-2 expressing cells
Authentication	Cell lines from ATCC were not authenticated.<br>HEK293T-hACE-2 was examined for hACE-2 expression on its surface by immunofluorescent staining
Mycoplasma contamination	All cell lines were tested negative for mycoplasma.
Commonly misidentified lines<br>(See ICLAC register)	No misidentified cell lines used in this study.

Animals and other research organisms

Policy information about studies involving animals; ARRIVE guidelines recommended for reporting animal research, and Sex and Gender in Research

Laboratory animals	Female K18-hACE2 mice (B6.Cg-Tg(K18-hACE2)2Prlmn/J), 7 weeks of age. (Procured from the Jackson Laboratory)<br>Female BALB/c mice (Mus musculus), 4-6 weeks of age. (Procured from Nomura Siam International, Bangkok, Thailand)
Wild animals	The study did not involve wild animals
Reporting on sex	Only female mice were used for this study. Male mice are more aggressive than female mice and increase the biosafety risk in the BSL3 during high titer intranasal challenge with virulent SARS-CoV-2.
Field-collected samples	The study did not involve sample collection from the field.
Ethics oversight	All studies were conducted under protocols approved by the Committees on Care of Laboratory Animal Faculty of Medicine, Chulalongkorn University (IACUC approval no. 007/2563), and the Armed Forces Research Institute of Medical Sciences, AFRIMS (IACUC approval no. PN20-06).

Note that full information on the approval of the study protocol must also be provided in the manuscript.
